# Teacher support and bullying victimization among Chinese migrant children: the mediating role of trait emotional intelligence and the moderating role of gender

**DOI:** 10.3389/fpsyg.2026.1901698

**Published:** 2026-08-11

**Authors:** Lihong Zeng, Xihua Chen

**Affiliations:** Wuyi University, Wuyishan, China

**Keywords:** bullying victimization, migrant children, moderated mediation, teacher support, trait emotional intelligence

## Abstract

Drawing on Bronfenbrenner's bioecological model and social role theory, this study examined whether trait emotional intelligence mediates the relationship between teacher support and bullying experiences among migrant children in China, and whether this indirect relationship varies by gender. A total of 451 upper-grade elementary school migrant children in China completed self-administered questionnaires. Through mediation and moderated mediation analyses, the results showed that teacher support was negatively correlated with experiences of bullying. Trait emotional intelligence statistically mediated the association between teacher support and bullying victimization. Furthermore, gender moderated the relationship between trait emotional intelligence and bullying victimization: the negative correlation was significant among boys but not among girls. Gender did not significantly moderate the direct relationship between teacher support and bullying victimization. These findings contribute to understanding how school-level relational support and children's emotional resources are jointly associated with bullying victimization among migrant children, and provide practical insights for schools serving migrant children in implementing anti-bullying interventions.

## Introduction

School bullying is widely recognized as a significant threat to children's mental health and subjective wellbeing (Yoon and Kerber, 2003; UNESCO, 2019). Bullying victimization in schools refers to repeated and deliberate attacks on defenseless victims by individuals and groups in schools, causing physical and mental harm to the victims (Olweus, 1993). Some studies indicate that chronic victimization elevates risks of suicidal ideation, depressive symptoms, and aggressive retaliation (Hodges and Perry, 1999; Arslan et al., 2021; Moore et al., 2017). Bullying victimization can also have a medium-to-long-term impact on the victim's life, which cause mistrust of others and self-concept problems. Aggressors may exhibit maladaptive behavior and eventually become involved in criminal behavior (Goodwin et al., 2019; Nickerson, 2019). Beyond perpetrators and victims, bystanders also suffer, without intervention, they may develop anxiety, self-blame, or themselves become perpetrators to avoid victimization (Lereya et al., 2015).

Although these findings generally highlight the serious consequences of being bullied, existing research has primarily focused on the general student population. This has left a critical gap in understanding how protective factors at the school level and individual emotional resources interact among migrant children, a group facing the dual risks of social marginalization and emotional vulnerability.

To understand bullying victimization among migrant children, this study draws primarily on Bronfenbrenner's bioecological model (Bronfenbrenner and Morris, 2006) and Social Role Theory (Eagly and Wood, 2012). Previous studies have examined the direct link between teacher support and bullying victimization, but evidence regarding the emotional mechanisms [e.g., Trait emotional intelligence (Trait EI)] that mediate this relationship remains limited and inconsistent. Some research suggests that teacher support enhances students' emotional competence, thereby reducing victimization risk (Kokkinos and Kipritsi, 2011; Zeng et al., 2023); other studies report non-significant mediation effects (Wu et al., 2023), highlighting the need for a clearer theoretical and empirical inquiry. The bioecological model conceptualizes development as emerging from the interaction between person characteristics and proximal environmental contexts. Within this framework, teacher support is conceptualized as a proximal school microsystem resource, whereas Trait EI is conceptualized as a person-level emotional resource that may help children interpret, regulate, and respond to stressful peer interactions. While parental and peer support also operate within the microsystem, the present study focuses specifically on teacher support for three reasons. First, migrant children often experience disrupted family routines and weakened peer networks following relocation (Li and Hu, 2020; National Bureau of Statistics of China, 2025), More importantly, they face unique challenges, such as social exclusion by local peers, difficulties adapting to school life due to differences in curriculum and dialects, discrimination from teachers and classmates, stress resulting from the transition between rural and urban environments, and identity conflicts between the culture of their hometown and that of the city where they live (Zhang et al., 2020).

These intertwined adversities make migrant children particularly vulnerable to bullying; therefore, although the school environment provides a relatively stable space for development, it also requires targeted, system-based interventions. Rendering the school context a comparatively stable developmental niche. Second, teachers constitute the most policy-actionable agents within this niche, offering clearer implications for school-based intervention. Third, focusing on a single microsystem agent allows for a more precise examination of the proposed emotional mechanism. Unlike previous studies, this research integrates Bronfenbrenner's bioecological model with social role theory to examine a moderated mediation framework. Specifically, it investigates how Trait EI mediates the relationship between perceived teacher support and bullying victimization, while exploring gender as a moderator in the link between teacher support and Trait EI This theoretical integration is innovative as it simultaneously addresses several critical research gaps. It provides much-needed empirical evidence of the emotional mechanisms underlying bullying dynamics among migrant children, and it helps clarify previous inconsistent findings regarding the mediating role of Trait EI. Furthermore, it incorporates gender as a crucial contextual moderator of this emotional pathway, an aspect that has been largely overlooked in prior literature.

The bioecological model posits that external context must interact with internal “Person” characteristics to shape developmental outcomes. Trait EI is a constellation of emotion-related self-perceptions and dispositions comprising the affective aspects of personality (Petrides, 2009; Kokkinos and Kipritsi, 2011). Therefore, we selected trait EI as the key mediating variable rather than broader cognitive or self-evaluation constructs (such as general self-efficacy), because bullying victimization is an emotionally charged interpersonal experience closely linked to emotional perception and regulation. Trait EI may represent an emotional resource through which perceived teacher support is associated with more adaptive responses to bullying-related peer difficulties. Complementing this, Social Role Theory (Eagly and Wood, 2012) highlights how societal gender norms shape emotional processing and social interactions, justifying the inclusion of gender as a key moderator. By specifying this novel moderated mediation framework, the study not only offers a theoretically grounded explanation of how teacher support may reduce victimization through emotional pathways, but also provides actionable insights for school- based interventions targeting migrant children's unique socio- emotional needs.

Because the present study relied on cross-sectional data, the proposed pathways should be interpreted as theory-driven statistical associations rather than evidence of causal mediation (Kline, 2015; Pirlott and MacKinnon, 2016). Nevertheless, the model offers a theoretically grounded framework for examining how school-based support and children's emotional resources may be jointly associated with bullying victimization among migrant children and it may inform future longitudinal and intervention studies.

## Literature review

### Teacher support and bullying victimization

Within ecological systems theory, the school is a central microsystem in which repeated interactions with significant adults shape children's developmental outcomes (Bronfenbrenner, 1979). Because migrant children typically rely on a narrower social support network than their local peers, perceived teacher support may carry disproportionate protective weight in this subgroup. Teacher support generally refers to students' perceptions that teachers are caring, respectful, available, and responsive to their academic and emotional needs. In a supportive classroom environment, students may feel safer reporting problems, more strongly connected to school, and less vulnerable to peer aggression.

This perspective may be especially relevant for migrant children. Compared with non-migrant peers, migrant children often face relocation stress, social exclusion, and reduced access to stable support networks (Li and Hu, 2020; Xie and Zhang, 2021). Under such conditions, supportive teachers may serve as a particularly salient protective resource within the school microsystem. Empirical studies have consistently documented a negative association between perceived teacher support and bullying victimization (Yang et al., 2022; Ren et al., 2023; Zeng et al., 2023). In addition, teachers' intervention-related beliefs and behaviors have been linked to lower levels of bullying victimization in classrooms (Troop-Gordon and Ladd, 2015; Demol et al., 2020; Veenstra et al., 2014; Yoon et al., 2021). Based on ecological systems theory and this empirical literature, we proposed the following hypothesis:

Hypothesis 1: Teacher support may be negatively associated with bullying victimization among migrant children.

### The mediating role of trait EI

Ecological systems theory also implies that environmental influences may become internalized as enduring personal resources that help children adapt to future challenges. In the present study, we consider Trait EI as one such resource. In peer conflict contexts, Trait EI facilitates adaptive emotion regulation, help-seeking, and de-escalation, all of which reduce exposure to recurrent victimization (Yang et al., 2022). Teacher support may be linked to Trait EI because supportive teachers can model emotion regulation, validate students' feelings, and create interactional contexts in which children learn to identify and express emotions appropriately. Prior studies suggest that positive teacher-student relationships are associated with children's social-emotional competence and emotional adjustment (Cheng et al., 2025; Zeng et al., 2023). In turn, higher Trait EI has been associated with lower levels of victimization and better psychosocial adaptation in peer contexts (Kokkinos and Kipritsi, 2011; Quintana-Orts et al., 2019). Emotional regulation and cooperation skills were found to be protective factors associated with lower levels of bullying in both traditional and online settings (Li et al., 2026).

For migrant children, this pathway may be especially relevant. Because migration can involve stress, stigma, reduced social support, and difficulties adapting to new school environments (Lin et al., 2009; Shen et al., 2015; Yang et al., 2022). Against such adverse developmental contexts, emotional self-regulation functions as a critical psychological pathway that connects positive school resources (i.e., teacher support) to reduced risk of bullying victimization and peer maladjustment (Jennings and Greenberg, 2009; Quintana-Orts et al., 2019). Building on this theoretical and empirical foundation, the present study further tests whether Trait EI statistically mediates the association between perceived teacher support and bullying victimization among migrant children. Thus, we hypothesized:

Hypothesis 2: Trait EI may mediate the association between teacher support and bullying victimization.

### The moderating role of gender

To explain possible differences in the strength of these associations, we draw on social role theory (Walker, 1999; Eagly and Wood, 2012). This theory posits that boys and girls are exposed to somewhat different social expectations concerning emotional expression, interpersonal orientation, assertiveness, and help-seeking. These gendered expectations may shape how internal emotional resources function in peer contexts. For example, boys may be less likely to seek adult help when victimized because norms of toughness and independence discourage the expression of vulnerability. Under such circumstances, Trait EI may become especially important because it may help boys regulate distress, respond more flexibly in conflict situations, and seek assistance more strategically. For girls, by contrast, bullying victimization often takes more relational forms embedded within social networks (Rose and Rudolph, 2006), meaning that the protective role of individual emotional resources may depend more strongly on contextual factors beyond Trait EI alone.

**Figure 1:**
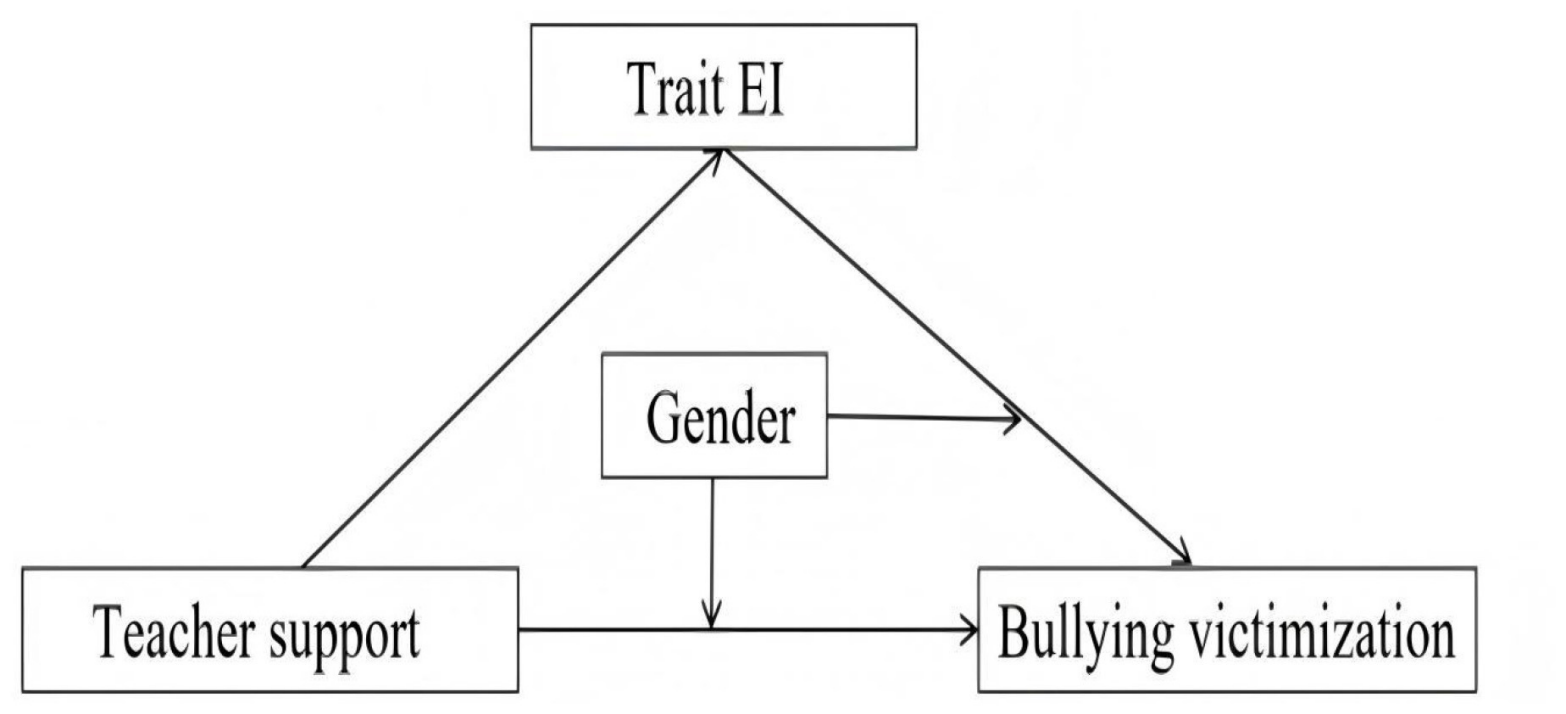
The moderated mediation model.

Prior findings on gender differences in bullying-related processes are mixed, and evidence on gendered effects of multiple social supports is inconsistent (Rueger et al., 2010); some studies suggest that the association between emotional competencies and victimization may be stronger for boys than for girls (Peachey et al., 2017; Zhao et al., 2021). Evidence regarding whether gender moderates the direct association between teacher support and victimization is less consistent. Therefore, we specified a hypothesis as follows:

Hypothesis 3: Gender may moderate the direct relationship between teacher support and bullying victimization, as well as the relationship between Trait EI and bullying victimization; however, no specific assumptions are made regarding the direction or magnitude of these moderating effects. The proposed moderated mediation model is illustrated in Figure 1.

## Methods

### Participants and procedure

This study utilized a quantitative, cross-sectional survey design. Participants were recruited using convenience cluster sampling from five public elementary schools in northern Fujian Province. This specific sampling method was selected because purely random sampling was logistically unfeasible due to administrative constraints, and sampling intact classrooms minimized disruption to regular school routines. All eligible fourth- to sixth-grade migrant children in selected classes were invited to participate. Of the 482 questionnaires collected, 31 (representing 6.4% of the initial sample) were excluded. Specifically, following standard data cleaning procedures, cases were removed using listwise deletion if they had excessive missing responses (defined as missing more than 10% of items), patterned responding. The final sample comprised 451 migrant children (255 boys, 56.7%; 196 girls, 43.3%), including 144 fourth-graders (31.9%), 106 fifth-graders (23.5%), and 201 sixth-graders (44.6%).

Regarding only-child status, 64 participants (14.2%) were only children, while 387 (85.8%) had siblings. Participants were recruited through their schools and asked to complete a questionnaire. Drawing on previous research on migration in China (Tan, 2010; Shen et al., 2015; Yang et al., 2022), this study defines ‘migrant children' as individuals whose place of residence differs from their registered domicile and who have been away from their registered domicile for more than 6 months.

This study was conducted in accordance with the guidelines approved by the Ethics Committee of Wuyi University (Approval No. 2025001). Written informed consent was obtained from parents/legal guardians, and verbal assent was obtained from all participating children prior to data collection. Schools also provided administrative approval. Data collection was conducted in familiar, quiet, and comfortable school classrooms by trained research assistants. To ensure an appropriate environment and minimize reporting bias, school teachers were asked not to be present in the room during the questionnaire administration. To ensure participant confidentiality throughout the study, the survey was completely anonymous, and all collected data were stored securely on password-protected computers accessible only to the research team.

## Measures

### Trait emotional intelligence questionnaire–child short form (TEIQue-CSF)

Trait EI was measured by the TEIQue-CSF (Mavroveli et al., 2009, 2008). The TEIQue-CSF was translated into Chinese following standard cross-cultural translation procedures (Brislin, 1970) and underwent cultural adaptation. First, two bilingual researchers independently translated the original English scale items into Chinese. Subsequently, a third bilingual expert back-translated the Chinese version into English, and any discrepancies were resolved through discussion with the original author. The preliminary Chinese version was pilot-tested on 30 migrant children to assess item clarity and cultural relevance, and the wording was fine-tuned accordingly. The final version was subsequently used in the main study. The 36 items measured nine dimensions. The questionnaire was scored using a five-point scale (1 = *strongly disagree*, 5 = *strongly agree*), where higher scores indicated better Trait EI. In this study, the TEIQue-CSF has three dimensions: affective disposition (e.g., I often feel angry), self-motivation (e.g., I don't like studying hard), and emotion perception (e.g., It's easy for me to understand how I feel). In the current study, the Chinese version of the TEIQue-CSF exhibited a high degree of internal consistency (α = 0.86). AMOS 22.0 was used to perform confirmatory factor analysis (CFA). The results of the CFA showed a good fit of the model, with the following findings: χ^2^/*df* = 2.222, *GFI* = 0.955, *NFI* = 0.913, *TLI* = 0.936, *CFI* = 0.950, *RMSEA* = 0.051, and *SRMR* = 0.045. These results indicated that the scale exhibited good reliability and validity.

### Bullying victimization

Our research employed the revised Chinese version of the Delaware Bullying Victimization (Student) Scale, a tool developed by Xie and Wei (2018). The original scale consisted of 16 items and encompassed four dimensions (verbal bullying, physical bullying, relationship bullying, and cyber bullying). The cyberbullying subscale was excluded because preliminary consultation with participating schools indicated that mobile phone use was prohibited during school hours and that most upper-primary students had limited independent device access at home. For the purpose of this research, we selected three elements: verbal bullying (e.g., Some of my classmates uttered very unkind words to me), physical bullying (e.g., I've been violently punched or kicked), and relational bullying (e.g., My classmates ostracized me from some events, and it made me feel bad). It is a six-point rating scale ranging from 1 (*never*) to 6 (*every day*), totaling 12 items, where higher scores show more serious bullying. The Cronbach's alpha was 0.87 in the current study. We employed AMOS 22.0 to perform confirmatory factor analysis (CFA). The CFA results for bullying victimization demonstrated a good fit of the model, with the following findings: χ^2^*/df* = 3.699*, GFI* = *0*.957*, NFI* = 0.941*, TLI* = 0.931, *CFI* = *0*.956, *RMSEA* = 0.077, and *SRMR* = 0.041.

### Teacher support

Teacher support was measured using the Teacher Support Scale developed by Zhao et al. (2021), which includes 12 items across two subscales: emotional support and instrumental support. Representative items include “My teacher made me feel respected” for emotional support, and “Teachers care about my learning and teach me individually” for instrumental support. A five-point rating scale from 1 (*strongly disagree*) to 5 (*strongly agree*) was used, where higher scores indicate greater support from teachers. In this study, the Cronbach's alpha was 0.94. Amos 22.0 was used to perform Confirmatory Factor Analysis (CFA). The CFA for teacher support showed that the model fitted well: χ^2^*/df* = 3.844, *GFI* = 0.953, *NFI* = 0.969, *TLI* = 0.968, *CFI* = 0.977, *RMSEA* = 0.080, and *SRMR* = 0.027.

### Common method bias test

Since all study variables were measured via self-reported questionnaires, Harman's single-factor test was performed to test for potential common method bias (Podsakoff et al., 2003). All scale items were subjected to an unrotated exploratory factor analysis. The results revealed that the first extracted factor explained 18.55% of the total variance, which fell below the critical cutoff of 40%. This result indicates that severe common method bias did not contaminate the present dataset.

### Data analysis

Data analysis was conducted using SPSS and the PROCESS macro (Version 4.1; Hayes, 2022). First, descriptive statistics and Pearson's correlation coefficients were calculated for the main study variables. Subsequently, PROCESS Model 4 was used to conduct a mediation analysis to examine whether Trait EI statistically mediated the association between teacher support and experiences of bullying, while controlling for variables such as grade level, only-child status, and gender. PROCESS Model 15 was employed to conduct a moderated mediation analysis to test whether gender moderated the direct association between teacher support and victimization, as well as the association between Trait EI and victimization. Gender was coded as a dummy variable, with 0 representing male students and 1 representing female students. In the moderation-mediation model, both teacher support and Trait EI were mean-centered. All regression coefficients are presented as unstandardized coefficients *B*. Indirect effects were tested using 5,000 bootstrap samples; an effect was considered significant when the 95% bootstrap confidence interval did not include zero.

## Results

### Preliminary statistics

As shown in Table 1, teacher support was positively correlated with Trait EI (*r* = 0.579, *p* < 0.01), whereas bullying victimization was negatively correlated with both teacher support (*r* = −0.383, *p* < 0.01) and Trait EI (*r* = −0.333, *p* < 0.01), Gender was positively correlated with Trait EI but was not significantly correlated with teacher support or bullying victimization.

**Table 1:** Descriptive statistics and correlations between variables.

Variables	*M*	*SD*	1	2	3
Trait EI	4.350	0.758	1		
TS	3.956	0.629	0.579^**^	1	
BV	1.463	0.509	−0.333^**^	−0.383^**^	1

*N* = 451; ^*^p < 0.05, ^**^p < 0.01; TS, Teacher support; BV, Bullying victimization.

Correlations involving demographic variables are reported in the expanded correlation matrix.

### Testing for the mediation model

To examine the mediating role of Trait EI, PROCESS Model 4 was conducted with grade, only-child status, and gender included as covariates. The results indicated that teacher support was significantly associated with lower bullying victimization before Trait EI was entered into the model (*B* = −0.277, *p* < 0.001).

Results presented in Table 2 demonstrate that, with the inclusion of Trait EI, teacher support was also positively associated with Trait EI (*B* = 0.519, *p* < 0.001), and Trait EI was negatively associated with bullying victimization when teacher support was controlled (*B* = −0.103, *p* < 0.01). Moreover, we employed the bootstrapping method to compute the standard of the associated effects and the confidence intervals among the effects. The results revealed a significant mediating effect of Trait EI, with an indirect effect of −0.054, accounting for 19.36% of the total effect. The 95% confidence interval for this indirect effect was [−0.103, −0.008]. Moreover, teacher support had a significant direct effect on the outcome, with a coefficient of −0.22, representing 80.64% of the total effect and a 95% confidence interval of [−0.309, −0.138].

**Table 2:** The mediation model of Trait EI between teacher support and bullying victimization.

Predictors	Model 1 (BV)		Model 2 (Trait EI)		Model 3 (BV)	
	*B*	*SE*	*B*	*SE*	*B*	*SE*
TS	−0.277^***^	0.040	0.519^***^	0.050	−0.223^***^	0.044
Trait EI					−0.103^**^	0.037
*R* ^2^	0.169		0.413		0.183	
*F*	22.731^***^		78.344^***^		19.993^***^	

*N* = 451; ^*^p < 0.05, ^**^p < 0.01, ^***^p < 0.001; Bootstrap sample size = 5,000; Unstandardized coefficients are reported. Grade, only-child status, and gender were controlled.

The results show that the indirect effects in all models were statistically significant, with 95% confidence intervals excluding zero (see Table 3), which means that the relations between teacher support and bullying victimization were mediated by Trait EI. The mediation effect represented 19.36% of the total effects.

**Table 3:** Bootstrapping indirect effect and 95% confidence interval (CI) for the mediation model.

Variables	Estimated effect	SE	95% CI	Ratio to total effect (%)
Total effect	−0.277	0.040	[−0.355, −0.199]	—
Direct effect	−0.223	0.044	[−0.309, −0.138]	80.64
Indirect effect	−0.054	0.025	[−0.103, −0.008]	19.36

*N* = 451. Bootstrap sample size = 5,000. CI, confidence interval.

### Testing for the moderated mediation model

Analyses were performed using Model 15 in the PROCESS macro, with teacher support and Trait EI (mean-centered) included as independent variables. To examine the moderating role of gender, interaction terms were created by multiplying gender with teacher support and Trait EI (i.e., teacher support × gender; Trait EI × gender). The socio-demographic variables (grade and only-child status) were controlled for in the analysis.

The results indicated that gender moderated the relationship between Trait EI and bullying victimization (see Table 4). Specifically, the interaction term between gender and Trait EI significantly predicted bullying victimization (*B* = 0.306, *p* < 0.001), suggesting that the relationship between Trait EI and bullying victimization differed by gender. In contrast, the interaction term between gender and teacher support was not significant (*B* = 0.082, *p* = 0.350), suggesting that gender did not moderate the relationship between teacher support and bullying victimization.

**Table 4:** Moderated mediation effect test.

Predictors	BV		
	*B*	*SE*	*t*
Gender	−1.628	0.365	−4.461
TS	−0.221	0.057	−3.862^***^
Trait EI	−0.326	0.066	−4.927^***^
TS × Gender	0.082	0.088	0.935
Trait EI × Gender	0.306	0.080	3.843^***^
*R* ^2^	0.225		
*F*	18.371		

*N* = 451, ^***^p < 0.001. Teacher support and Trait EI were mean-centered. Gender was coded as 0 = boys and 1 = girls. Grade and only-child status were controlled. Unstandardized coefficients are reported.

**Figure 2:**
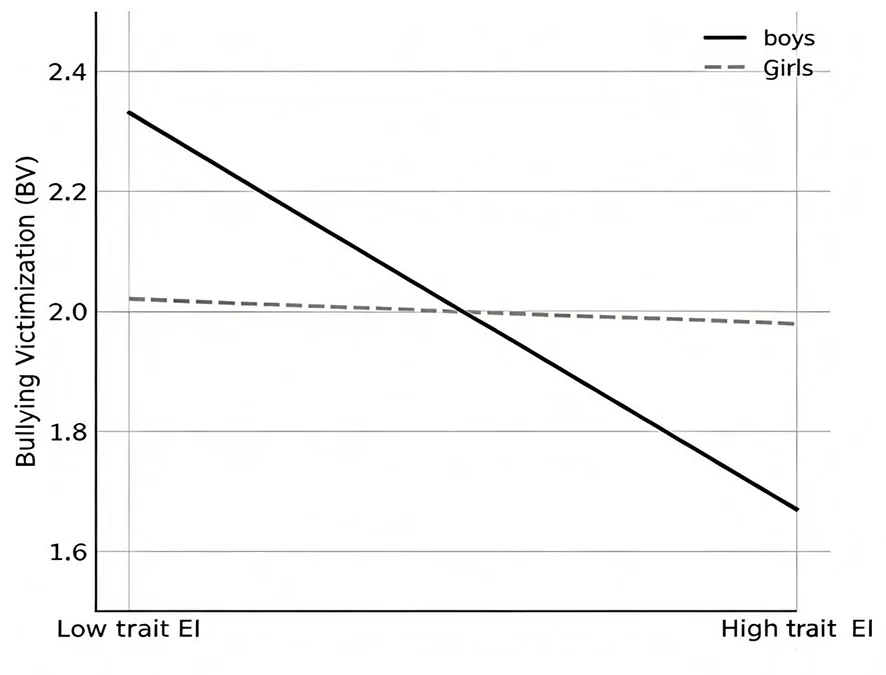
The relationship between Trait EI and bullying victimization.

To further explore the moderating effect of gender on the path from Trait EI to bullying victimization (see Table 5). For boys, the direct effect of teacher support on bullying victimization was significant (*B* = −0.221, *p* < 0.001). Meanwhile, the indirect association between teacher support and bullying victimization through Trait EI was also significant (*B* = −0.228, 95% CI [−0.330, −0.131]), with the confidence interval excluding zero. For girls, the direct effect of teacher support on bullying victimization reached significance (*B* = −0.139, *p* < 0.05). In contrast, the indirect association via Trait EI was not significant (*B* = −0.015, 95% CI [−0.096, 0.062]), as the confidence interval contained zero. These findings indicate that the indirect pathway from teacher support to bullying victimization through Trait EI was significant only for boys and not for girls. Simple slope analyses were conducted (see Figure 2). For boys, bullying victimization exhibited a significant negative association with Trait EI, *b* = −0.33, *SE* = 0.066, *t*_(443)_ = −4.93, *p* < 0.001, 95% CI [−0.456, −0.196]. This indicates that Trait EI served as a strong protective factor against bullying victimization for boys. For girls, however, the effect of Trait EI was not significant, *b* = −0.02, *SE* = 0.044, *t*_(443)_ = −0.47, *p* = 0.636, 95% CI [−0.107, 0.066]. The difference between the two simple slopes was supported by a significant Trait EI × gender interaction, Δ*R*^2^ = 0.026, *F*_(1, 443)_ = 14.77, *p* < 0.001. Therefore, H3 was partially supported.

**Table 5:** Conditional direct and indirect effects of teacher support on bullying victimization by gender.

Gender	Indirect effect			Direct effect		
	*B*	*BootSE*	95% CI	*B*	*BootSE*	95% CI
Boys	−0.228	0.051	[−0.330, −0.131]	−0.221	0.057	[−0.334, −0.109]
Girls	−0.015	0.040	[−0.096, 0.062]	−0.139	0.066	[−0.270, −0.009]

## Discussion

This study examined whether Trait EI statistically mediates the relationship between teacher support and bullying victimization among migrant children in China, and whether this mediating process varies by gender. Overall, the findings were largely consistent with the proposed theoretical framework. Teacher support, as a key resource within the proximal school microsystem, was negatively associated with bullying victimization. Trait EI statistically explained part of this association. Furthermore, gender moderated the relationship between Trait EI and bullying victimization: this negative correlation was significant among boys but not among girls. However, gender did not significantly moderate the direct relationship between teacher support and bullying victimization. Therefore, the moderated mediation hypothesis was only partially supported.

### The mediating role of trait emotional intelligence

The results indicate that teacher support is positively correlated with Trait EI, while Trait EI is negatively correlated with bullying victimization. The indirect effect of teacher support on bullying victimization via Trait EI is significant, supporting the proposed mediating hypothesis (Hypotheses 1 and 2). This pattern is consistent with previous research, which has shown that supportive teacher-student relationships are associated with children's social-emotional competencies and more adaptive peer interactions (Longobardi et al., 2021; Zeng et al., 2023). This pattern is also consistent with findings indicating that emotional intelligence is associated with lower levels of bullying victimization and better psychosocial adjustment in peer contexts (Kokkinos and Kipritsi, 2011; Quintana-Orts et al., 2019).

From the perspective of Bronfenbrenner's bioecological model, teacher support can be viewed as a critical microsystem resource (Bronfenbrenner and Morris, 2006). Supportive teachers provide emotional warmth, respect, and classroom order, and intervene promptly when peer relationship issues arise. For migrant children, who often face profound stressors arising from cultural mobility, frequent school transitions, weak peer networks, and acute risks of social exclusion, this supportive adult presence is particularly important (Xie and Zhang, 2021; Yang et al., 2022). Due to China's Hukou (household registration) system, migrant children frequently encounter institutional stigma, perceived discrimination, and difficulties in cultural adaptation within urban schools, which can threaten their social identity and increase their vulnerability to peer marginalization. When students perceive their teachers as caring and responsive, they feel safer seeking help, expressing distress, and navigating the complexities of cultural adaptation and identity negotiation. This perspective aligns with research findings indicating that positive teacher-student relationships are closely linked to children's school adjustment and subsequent social-emotional development (Hamre and Pianta, 2001; Longobardi et al., 2021).

Importantly, according to the Trait EI theory (Petrides, 2009), Trait EI is defined as a construct located at a lower level of the personality hierarchy, comprising emotional self-awareness and emotional self-efficacy; it is the primary psychological mechanism that explains why teacher support can reduce the incidence of bullying. Supportive teacher-student interactions provide children with opportunities to observe, practice, and internalize affective self-efficacy. Teachers who lead by example—demonstrating empathy, acknowledging students' emotions, and guiding them in resolving conflicts—may help foster children's emotional self-awareness and perceived capacity to regulate negative affect. This explanation aligns with the prosocial classroom model, which emphasizes the role of teachers' emotional and interpersonal competencies in shaping classroom climate and promoting student adjustment (Jennings and Greenberg, 2009). For migrant children who may face uncertainty and stress while adapting to a new school environment, stronger emotional awareness and regulation skills are particularly important for managing peer interactions and reducing the risk of being bullied.

### The moderating role of gender

The results of the moderation analysis indicate that gender does not significantly moderate the direct relationship between teacher support and bullying victimization. In other words, the negative correlation between teacher support and bullying victimization is similar among both boys and girls. This finding suggests that, regardless of gender, teacher support may serve as a school resource that provides broad protection for migrant children. Supportive teachers are able to establish clearer classroom norms, curb bullying behavior, and increase students' willingness to report peer bullying. These processes may benefit both boys and girls.

However, gender played a significant moderating role in the relationship between Trait EI and experiences of bullying. Specifically, there was a significant negative correlation between Trait EI and experiences of bullying among boys, whereas this association was not significant among girls. This finding suggests that, in the context of bullying, Trait EI may serve as a more prominent protective resource for boys. Indicators of mediational significance further suggest that the indirect effect of teacher support on bullying victimization via Trait EI is stronger among boys than among girls.

This result can be explained by social role theory (Eagly and Wood, 2012). During socialization, boys are often expected to demonstrate resilience, independence, and emotional restraint. Such gendered expectations may make boys reluctant to openly express vulnerability or seek adult help when facing peer aggression. In this context, boys with higher Trait EI may be better able to identify their own emotional states, regulate distressing emotions, interpret social cues, and select more adaptive coping strategies. Therefore, Trait EI may play a particularly important protective role for boys.

It is worth noting that our findings intersect with the complex and sometimes contradictory literature on the relationship between Trait EI and peer bullying across genders. Some early studies suggest that emotional intelligence may be associated with lower levels of victimization, particularly among boys (Peachey et al., 2017). However, the findings of this study differ However, our findings differ from those of several notable studies. These studies either reported consistent associations between emotional intelligence and bullying behavior across gender groups (Lomas et al., 2012) or reported conflicting patterns, namely that emotional competence served as a significantly stronger buffer against bullying for girls but not for boys (Rueger et al., 2016). Furthermore, some empirical studies have even reported inconsistent or statistically insignificant associations between high emotional intelligence and lower levels of peer bullying, suggesting that under certain social evaluation conditions, heightened emotional awareness may make adolescents unusually sensitive to subtle forms of exclusion but does not necessarily equip them with the ability to prevent relational aggression (Mavroveli et al., 2008).

Most importantly, these theoretical and empirical discrepancies can be attributed to the unique context of our sample: children of migrants moving from rural to urban areas in China. Boys find themselves at the intersection of traditional masculine socialization (which emphasizes resilience) and the vulnerabilities of migration (facing greater pressure to assimilate and the need to overcome social stigma). For these boys, expressing vulnerability leads to double stigmatization, making Trait EI an indispensable yet scarce psychological buffer mechanism for self-regulation and conflict resolution. In contrast, the lack of a significant association between Trait EI and bullying among girls reflects that the peer relationship difficulties they face are overwhelmingly interpersonal in nature. Bullying experiences among girls may involve exclusion, manipulation of friendships, rumor-mongering, and alliance-based peer interactions. Such relational forms of bullying may depend more on peer group structures, social status, and classroom social network processes than on individual emotional resources alone. This explanation is consistent with previous research indicating gender differences in peer relationship processes and forms of bullying (Zhao and Zhou, 2016; Rose and Rudolph, 2006; Smith et al., 2019). It also aligns with research indicating that relational victimization is closely associated with peer acceptance, exclusion, popularity, and friendship characteristics (Casper et al., 2020). Therefore, for girls, Trait EI alone may not be sufficient to reduce the risk of victimization unless it is accompanied by supportive peer relationships and inclusive classroom norms.

These findings have practical implications for schools serving migrant children. First, strengthening teacher support should be viewed as a universal prevention strategy. Teachers can reduce instances of bullying by creating emotionally safe classroom environments, actively mitigating urban-rural cultural discrimination, responding consistently to bullying incidents, and building relationships of mutual trust with students. When migrant children encounter difficulties related to cultural adaptation or peer exclusion, teachers should demonstrate respect, acceptance, and care for their students' emotions. Such support not only deepens the teacher-student bond, increases students' willingness to share their problems, and fosters a more harmonious classroom atmosphere, but also reduces the incidence of bullying (Bergin and Bergin, 2009; Zeng et al., 2023). Second, intervention strategies may need to be gender-sensitive and culturally responsive. For migrant boys, programs can focus on helping students translate emotional awareness and regulation into specific coping behaviors, such as seeking help, communicating confidently, and de-escalating conflicts. For migrant girls, interventions may need to go beyond the scope of individual emotional skills and place greater emphasis on restructuring peer group norms, cultivating intercultural friendship quality, dismantling relational bullying networks, and fostering collective classroom inclusivity.

### Limitations and future directions

This study acknowledges several limitations that warrant attention. First, the cross-sectional nature of the data precludes causal inferences. As Kline (2015) emphasizes, statistical mediation should not be equated with causal mediation. To address this methodological limitation related to research design, future studies should shift from static cross-sectional methods to longitudinal Cross-Lagged Panel Models (CLPM) or Latent Growth Curve Models (LGCM). Tracking changes in teacher support, Trait EI, and bullying victimization across multiple time points—particularly during the critical transition period when immigrant children first enter urban schools—will help clarify their developmental trajectories and temporal sequences. Furthermore, conducting school-based randomized controlled trials (RCTs) to implement experimental interventions involving teacher support strategies will allow for rigorous testing of the causal hypotheses within our moderating-mediation framework (Pirlott and MacKinnon, 2016).

Second, all study variables relied on student self-reports, which may lead to common-method bias and subjective reporting distortions. Although Harman's one-factor test suggests that such bias is unlikely to be severe, future research should incorporate multi-source data. Integrating sociometric peer nominations, objective behavioral observations, and teacher ratings of peer interactions would provide a much more reliable capture of relational victimization, cultural discrimination, and classroom dynamics among migrant children.

Third, regarding the sampling strategy, our convenience cluster sampling in northern Fujian limits the generalizability of the findings to other economically diverse regions in China.

Future research should employ stratified random sampling to capture migrant children across different destinations. The study design should include a comparison group comprising peers from both urban and rural areas. Rigorous analytical methods, such as multi-group structural equation modeling (SEM), should be employed, along with tests of measurement invariance across gender and migration status.

### Conclusion

By integrating Bronfenbrenner's bioecological model, Trait EI Theory, and social role theory, this study contributes to the existing literature on bullying among migrant children in China. The results indicate that teacher support is negatively correlated with bullying, and that Trait EI statistically mediates this relationship by empowering children with emotional self-efficacy to navigate adaptation stress. Furthermore, gender moderates the relationship between Trait EI and bullying: a significant negative correlation was observed among boys, whereas no such correlation was found among girls. These findings suggest that school-based anti-bullying interventions targeting migrant children would be more effective if they simultaneously strengthen teacher-student relationships and incorporate gender-sensitive strategies for developing emotional and interpersonal skills. However, as this study relies on cross-sectional data, longitudinal and intervention studies are needed to further test the temporal sequence and causal relationship assumptions implied by the proposed model.

## Data Availability

The raw data supporting the conclusions of this article will be made available by the authors, without undue reservation.
